# Farmers’ Wives Overtaxed

**Published:** 1863-02

**Authors:** 


### FARMERS' WIVES OVERTAXED.

THERE is scarcely any lot in life, in this country, which promises so much quiet enjoyment, such uniform health and uninterrupted prosperity, as that of a gentleman farmer's wife; of a man who has a well-improved, well-stocked plantation, all paid for, with no indebtedness, and a sufficient surplus of money always at command, to meet emergencies, and to take advantage of those circumstances of times and seasons and changing conditions which are constantly presenting themselves. Such a woman is incomparably more certain of living in quiet comfort to a good old age than the wife of a merchant-prince, or one of the money-kings of Wall street; who, although they may clear thousands in a day, do, nevertheless, in multitudes of cases, die in poverty, leaving their wives and daughters to the sad heritage of being slighted and forgotten by those who once were made happy by their smiles; and to pine away in tears and destitution. On the other hand, it is often a sad lot indeed to be the wife of a farmer who begins married life by renting a piece of land or buying a "place" on credit, with the moth of "interest" feeding on the sweat of his face every moment of his existence.

The affectionate and steady interest, the laudable pride, and the self-denying devotion which wives have for the comfort, prosperity, and respectability of their husbands and children, is a proverb and a wonder in all civilized lands. There is an abnegation of self in this direction, as constant as the flow of time; so loving, so uncomplaining, so heroic, that if angels make note of mortal things, they may well look down in smiling admiration. But it is a melancholy and undeniable fact, that in millions of cases, that which challenges angelic admiration fails to be recognized or appreciated by the very men who are the incessant objects of these high, heroic virtues. In plain language, in the civilization of the latter half of the nineteenth century, a farmer's wife, as a too general rule, is a slave and a drudge; not of necessity, by design, but for want of that consideration, the very absence of which, in reference to the wife of a man's youth, is a crime. It is perhaps safe to say, that on three farms out of four, the wife works harder, endures more, than any other on the place; more than the husband, more than the "farm-hand," more than the "hired help" of the kitchen. Many a farmer speaks to his wife, habitually, in terms so imperious, so impatient, so petulant, that if repeated to the scullion of the kitchen, would be met with an indignant and speedy departure, or if to the man-help, would be answered with a stroke from the shoulder, which would send the churl reeling a rod away!

2. In another way a farmer inadvertently increases the hardships of his wife; that is, by speaking to her or treating her disrespectfully in the presence of the servants or children. The man is naturally the ruling spirit of the household, and if he fails to show to his wife, on all occasions, that tenderness, affection, and respect, which is her just due, it is instantly noted on the part of menials, and children too, and they very easily glide into the same vice, and interpret it as an encouragement to slight her authority, to undervalue her judgment and to lower that high standard of respect, which of right belongs to her. And as the wife has the servants and children always about her, and is under the necessity of giving hourly instructions, the want of fidelity and promptness to these, is sufficient to derange the whole household, and utterly thwart that regularity and system, without which there is no domestic enjoyment, and but little thrift on the farm.

The indisputable truth is, that there is no other item of superior, or perhaps equal importance, in the happy and profitable management of any farm, great or small, than that every person on it should be made to understand, that deference and respect and prompt and faithful obedience, should be paid, under all circumstances, to the wife, the mother, and the mistress; the larger the farm, the greater interests there are at stake. If poor, then the less ability is there to run the risk or losses which are certain to occur in the failure of proper obedience. An illustration: a tardy meal infallibly ruffles the temper of the workmen, and too often of the husband; yet all the wife's orders were given in time; but the boy has lagged in bringing wood; or the cook failed to put her loaf to bake in season, because they did not fear the mistress, and the master was known not to be very particular to enforce his wife's authority. If by these causes a dinner is thrown back half an hour, it means on a good-sized farm a loss of time equivalent to the work of one hand a whole day; it means the very considerable difference between working pleasantly and grumblingly the remainder of the day; it means in harvest-time, in showery weather, the loss of loads of hay or grain.

3. Time and money and health, and even life itself, are not unfrequently lost by a want of promptitude on the part of the farmer in making repairs about the house, in procuring needed things in time, and failing to have those little conveniences which although their cost is even contemptible, are in a measure, practically invaluable. I was in a farmer's house one night; the wife and two daughters were plying their needles industriously by the light of a candle, the wick of which was frequently clipped off by a pair of scissors. I asked the husband why he did not buy a candle-snuffer. "Oh! the scissors are good enough." And yet he owned six hundred acres of fine grazing lands, and every inch paid for. I once called on an old friend, a man of education, and of a family, loved and honored all over his native State. The buildings were of brick, in the center of an inherited farm of several hundred acres. The house was supplied with the purest, coldest, and best water from a well in the yard; the facilities for obtaining which were a rope, one end of which was tied to a post, the other to an old tin pan, literally. The discomfort and unnecessary labor involved in these two cases, may be estimated by the reader at his leisure.

I know it to be the case, and have seen it on many Western farms, when firewood was wanted, a tree was cut down and hauled bodily to the door of the kitchen; and when it was all gone, another was drawn up to supply its place; giving the cook and the wife, green wood with which to kindle and keep up their fires.

There are thousands of farms in this country, where the spring which supplies all the water for drink and cooking, is from a quarter to more than half a mile distant from the house, and a "pailful" is brought at a time, involving five or ten miles' walking in a day, for months and years together; when a man in half a day could make a slide and with a fifty cent barrel could in half an hour deliver, at the door, enough to last the whole day. How many weeks of painful and expensive sickness; how many lives have been lost of wives and daughters and cooks, by being caught in a shower between the house and the spring, while in a state of perspiration or weakness, from working over the fire, can not be known; but that they may be numbered by thousands, will not be intelligently denied.

Many a time a pane of glass has been broken out, or a shingle has been blown from the roof, and the repair has not been made for weeks or many months together; and for want of it, have come agonizing neuralgias; or a child has waked up in the night with the croup, to get well only with a doctor's bill, which would have paid twenty times for the repair; even if a first-born has not died, to agonize a mother's heart to the latest hour of life; or the leak in the roof has remained, requiring the placing of a bucket or the washing of the floor at every rain; or the "spare bed" has been wetted and forgotten; some visitor or kind neighbor, or dear friend has been placed in it, to wake up to a fatal fever, as was the case with the great Lord Bacon.

4. Brutalities are thoughtlessly sometimes, and sometimes recklessly perpetrated by farmers on their wives as follows: a child or other member of the family is taken sick in the night; the necessary attention almost invariably falls on the wife, to be extended to a greater part, if not the whole night. Wearied with the previous day's duties, with those solicitudes which always attend sickness; with the responsibilities of the occasion, and a loss of requisite rest, the wife is many times expected to "see to breakfast" in the morning, as if nothing had happened. The husband goes to his work, soon becomes absorbed in it, and forgets all about the previous night's disturbance; meets his wife at the dinner-table; notices not the worn-out expression on her face; makes no inquiry as to her feelings; and if any thing on or about the table is not just exactly as it ought to be, it is noticed with a harshness which would be scarcely excusable if it had been brought about with a deliberate calculation.

The same thing occurs multitudes of times during the nursing periods of mothers; how many nights a mother's rest is broken half a dozen times by a restless, crying, or ailing infant, every mother and observant man knows; in such cases, the farmer goes into another room, and sleeps soundly until the morning, and yet, in too many cases, although this may be, and is repeated several nights in succession, the husband does not hesitate to wake his wife up with the information that it is nearly sunrise; the meaning of which is, that he expects her to get up and attend to her duties. No wonder that in many of our lunatic asylums, there are more farmers' wives than any other class; for there is no fact in medical science more positively ascertained, than that insufficient sleep is the most speedy and certain road to the madhouse; let no farmer then, let no mechanic, let no man, who has any human sympathy still left, allow his wife to be waked up in the morning, except from very urgent causes; and further, let them give every member of the household to understand that quietude about the premises is to be secured always until the wife leaves her chamber; thus having all the sleep which nature will take, the subsequent energy, cheerfulness, and activity which will follow, will more than compensate for the time required to "get her sleep out;" not only as to her own efficiency, but as to that of every other member of the household; for let it be remembered that a merry industry is contagious.

There are not a few farmers whose imperious wills will not brook the very slightest dereliction of duty on the part of any hand in their employ; and whose force of character is such, that every thing on the farm, outside the house, goes on like clockwork. They look to their wives to have similar management indoors; and are so swift to notice, even slight shortcomings, that at length their appearance at the family table has become inseparable from scenes of jarring, fault-finding, sneering, depreciating comparisons, if not of coarse vituperation, of which a savage might well be ashamed; and all this, simply from the failure to remember that they have done nothing to make the wife's authority in her domain, as imperative as their own; they make no account of the possible accidents of green wood to cook with; of an adverse wind which destroys the draft of the chimney; of the breaking down of the butcher's cart; or the failure of the baker to come in time; they never inquire if the grocer has not sent an inferior article, or an accident has befallen the stove or some cooking utensil. It is in such ways as these, and millions more like them, that the farmer's wife has her whole existence poisoned by those daily tortures which come from her husband's thoughtlessness, his inconsideration, his hard nature, or his downright stupidity. A wife naturally craves her husband's approbation. "Thy desire shall be to thy husband," is the language of Scripture; which, whatever may be the specific meaning of the quotation, certainly carries the idea that she looks up to him, with a yearning inexpressible, for comfort, for support, for smiles and sympathy; and when she does not get these, the whole world else is a waste of waters, or life a desert, as barren of sustenance as the great Sahara. But this is only half the sorrow; when in addition to this want of approbation and sympathy, there comes the thoughtless complaint; the remorseless and repeated fault-finding and the contemptuous gesture, when all was done that was possible under the circumstances—in the light of treatment like this, it is not a wonder that settled sadness and hopelessness is impressed on the face of many a farmer's wife, which is considered by the thoughtful physician, as the prelude to that early wasting away, which is the lot of many a virtuous and faithful and conscientious woman.

The attentive reader will not fail to have observed, that the derelictions adverted to on the part of farmer husbands, are not regarded necessarily as the result of a perverse nature; but rather in the main, from inconsideration or ignorance; but from whatever cause, the effect is an unmixed evil; and it is to be hoped, that our religious papers, and all agricultural publications, will persistently draw attention to these things, so as to excite a higher sentiment in this direction. It can be done and ought to be done; and high praise is justly due to the Honorable the Commissioner of the Agricultural Department, in that he has expressly desired, that an article should be written on the subject of the hardships and the unnecessary exposures of farmers' wives, to the end that information and instruction should be imparted in this direction; it is at once an evidence of a high and manly and generous nature.

There are some suggestions to be made with a view to lightening the load of farmers' wives, the propriety, the wisdom, and advantages of which, can not fail to be impressed on every intelligent mind.

1. A timely supply of all that is needed about a farmer's house and family, is of incalculable importance; and when it is considered that most of these things will cost less to get them in season, and also that a great deal of unnecessary labor can be avoided by so doing, it would seem only necessary to bring the fact distictly before the farmer's mind, to secure an immediate, an habitual and a life-long attention. The work necessary to keep a whole household in easily running order, is very largely curtailed by having every thing provided in time, and by taking advantage of those little domestic improvements devised by busy brains, and which are brought to public notice weekly in the columns of such papers as the *Scientific American* of New-York, for two dollars a year; in fact, it is of such a practical nature as to household matters that the writer has heretofore repeatedly suggested its patronage to the agricultural community, in spite of its repelling name to the more unlearned folk, who too often attach the idea of abstruseness, of difficulty of apprehension to any thing which has the word "scientific" attached to it; not knowing that it is the very essence of true science, its end and aim, to bring all truth to the easy comprehension of ordinary minds.

2. It requires less time and less labor to have the winter's wood for house-heating and cooking brought into the yard and piled up cozily under a shed or placed in a wood-house, in November, than to put it off until the ground is saturated with water, allowing the wheels to sink to the hub in mud; or until the snow is so deep as to make wheeling impossible.

3. It is incalculably better to have the potatoes and other vegetables gathered and placed in the cellar or in an outhouse near by in the early fall, so that the cook may get at them under cover; than to put it off week after week, until near Christmas; compelling the wife and servants once or twice every day, to leave a heated kitchen, and most likely with thin shoes, go to the garden with a tin pan and a hoe, to dig them out of the wet ground and bring them home in slosh or rain. The truth is, it perils the life of the hardiest persons, while working over the fire in cooking or washing, to go outside the door of the kitchen for an instant; a damp, raw wind may be blowing, which coming upon an inner garment, throws a chill or the clamminess of the grave over the whole body in an instant of time, to be followed by the reäction of fever, or fatal congestion of the lungs; or by making a single step in the mud, which is in tens of thousands of cases allowed to accumulate at the very door-sill, for want of a board or two, or a few flat stones, not a rod away.

4. No farmer's wife who is a mother ought to be allowed to do the washing of the family; it is perilous to any woman who has not a vigorous constitution. The farmer, if too poor to afford help for that purpose, had better exchange a day's work himself. There are several dangers to be avoided while at the tub—it requires a person to stand for hours at a time; this is a strain upon the young wife or mother, which is especially perilous—besides, the evaporation of heat from the arms, by being put in warm water and then raised in the air alternately, so rapidly cools the system that inflamation of the lungs is a very possible result; then, the labor of washing excites perspiration and induces fatigue; in this condition the body is so susceptible to taking cold that a few moments' rest in a chair, or exposure to a very slight draft of air, is quite enough to cause a chill, with results painful or even dangerous, according to the particular condition of the system at the time. No man has a right to risk his wife's health in this way, however poor, if he has vigorous health himself; and, if poor, he can not afford, for the five or six shillings which would pay for a day's washing, to risk his wife's health, her time for two or three weeks, and the incurring of a doctor's bill, which it may require painful economies for months to liquidate.

5. Every farmer owes it to himself, in a pecuniary point of view, and to his wife and children, as a matter of policy and affection, to provide the means early for clothing his household according to the seasons, so as to enable them to prepare against winter especially. Every winter garment should be completed by the first of November, ready to be put on when the first winter day comes. In multitudes of cases valuable lives have been lost to farmers' families by improvidence as to this point. Most special attention should be given to the under-clothing; that should be prepared first, and enough of it to have a change in case of an emergency or accident. Many farmers are even niggardly in furnishing their wives the means for such things; it is far wiser and safer to stint the members of his family in their food than in the timely and abundant supply of substantial under-clothing for winter wear. It would save an incalculable amount of hurry and its attendant vexations, and also of wearing anxiety, if farmers were to supply their wives with the necessary material for winter clothing as early as mid-summer. In this connection it would be well for farmers to learn a lesson of thrift from some of our long-headed city housewives; it is particularly the habit of the well-to-do, the forehanded, and the rich, by which they legally and rightfully get at least twenty per cent for their money; it is simply to purchase the main articles of clothing at the close of any season, to be made up and worn the corresponding season of next year. Merchants uniformly aim, especially in cities, to "close out" their stocks, for example, for the winter, at the end of winter or beginning of spring; they consider it profitable to sell out the remnant of their winter stock in March, at even less than cost, for on what they get for these remnants they make three profits, on the spring, the summer, and the fall goods; whereas, had they laid by their winter stock they would have had but one profit, from which would have to be deducted the yearly interest, storage, and insurance. Thus, by purchasing clothing materials six or eight months beforehand, the farmer not only saves from twenty to forty per cent of the first cost, but gives his wife the opportunity of working upon them at such odds and ends of time as would otherwise be unemployed in a measure, and would enable her also to have every thing done in a better manner, simply by having abundant time, thus avoiding haste, vexation, solicitude, and disappointment, for nothing so clouds a household as a sense of being behindhand and of the necessity of painful hurry and effort.

6. Few things will bring a more certain and happy reward to a farmer than for him to remember his wife is a social being, that she is not a machine, and therefore needs rest, and recreation, and change. No farmer will lose in the long run, either in money, health, or domestic comfort, enjoyment, and downright happiness, by allotting one afternoon in each week, from mid-day until bed-time, to visiting purposes. Let him, with the utmost cheerfulness and heartiness, leave his work, dress himself up, and take his wife to some pleasant neighbor's, friend's, or kinsman's house, for the express purpose of relaxation from the cares and toils of home, and for the interchange of friendly feelings and sentiments, and also as a means of securing that change of association, air, and food, and mode of preparation, which always wakes up the appetite, invigorates digestion, and imparts a new physical energy, at once delightful to see and to experience; all of which in turn tend to cultivate the mind, to nourish the affections, and to promote that breadth of view in relation to men and things which elevates, and expands, and ennobles, and without which the whole nature becomes so narrow, so contracted, so jejune and uninteresting, that both man and woman become but a shadow of what they ought to be.

7. Let the farmer never forget that his wife is his best friend, the most steadfast on earth, would do more for him in calamity, in misfortune, and sickness, than any other human being, and that on this account, to say nothing of the marriage vow, made before high heaven and before men, he owes to the wife of his bosom a consideration, a tenderness, a support, and a sympathy, which should put out of sight every feeling of profit and loss the very instant they come in collision with his wife's welfare as to her body, her mind, and her affections. No man will ever lose in the long run by so doing; he will not lose in time, will not lose in a dying hour, nor in that great and mysterious future which lies before all.

8. There are "seasons" in the life of women which, as to some of them, so affect her general system, and her mind also, as to commend them to our warmest sympathies, and which imperatively demand from the sterner sex the same patience, and forbearance, and tenderness which they themselves would want meted out to them if they were not of sound mind. At these times, some women, whose uniform good sense, propriety of deportment, and amiability of character command our admiration, become so irritable, fretful, complaining, quarrelsome, and unlovely as to almost drive their husbands mad; their conduct is so inexplicable, so changed, so perfectly causeless, that they are almost overcome with desperation, with discouragement, or indignant defiance of all rules of justice, of right, or of humanity. The ancients, noticing this to occur to some women for a few days in every month, gave it the appellation of "Lunacy," Luna being the Latin name for moon or monthly. Some women, at such times, are literally insane, without their right mind, and, as it is an infliction of nature, far be it from any husband, with the feelings of a man, to fail at such times to treat his wife with the same kind care, and extra tenderness, and pitying love that he would show to a demented only child. The skillful physician counsels in such cases the scrupulous avoidance of every word, or action, or even look which by any possibility could irritate the mind, excite the brain, or wound the sensibilities, and, as far as possible, to yield gracefully and good-naturedly to every whim and every caprice, to seem to control in nothing, to yield in all things; under these calming influences the mind sooner resumes its wonted rule, the heart gushes out in new loves and wakes up to a warmer affection than was ever known before. A misunderstanding of the case and an impatient resistance at all points has before now driven women to desperation, to a life-long hate, to suicide, or to a fate worse than all—to peer through the iron bars of a lunatic's cell for a long and miserable lifetime. Let every husband who has a human heart mature the subject well.

9. In these and other peculiar states of the system, arising from nervous derangement, women are sometimes childish, and various curious phenomena take place: there is an inability to speak for a moment or a month, the heart seems to "jump up in the mouth," or there is a terrible feeling of impending suffocation; at other times there are actual convulsions, or an uncontrollable bursting out into tears; these and other disagreeable phenomena are derisively and unfeelingly called "hysterics" or "nervousness," but they are no more unreal to the sufferer than are the pains of extraction for "nothing but the toothache." These symptoms are not unfrequently set down to the account of perverseness, when it should no more be done than to call it perversity to break out in uncontrollable grief at the sudden information of the death of the dearest friend on earth. The course of conduct to be pursued in cases of this kind is at once the dictate of science, of humanity, and of common-sense, it is to sympathize with and soothe the patient in all ways possible, until the excess of perturbation has passed away and the system calms down to its natural, even action.

10. Unless made otherwise by a vicious training, a woman is as naturally tasteful, tidy, and neat in herself and as to all her surroundings as the beautiful canary which bathes itself every morning, and will not be satisfied until each rebellious feather is compelled to take the shape and place which nature designed. It is nothing short of brutality to war against those pure, elevating, and refining instincts of a woman's better nature, and it is a husband's highest duty, his interest, and should be his pleasure and his pride to sympathize with his wife in the cultivation of these instincts, and to cheerfully afford her the necessary means, as far as he can do so consistently. No money is better spent on a farm or any where else than that which enables the wife to make herself, her children, her husband, and her house appear fully up to their circumstances. The consciousness of a torn or buttonless jacket or soiled dress degrades a boy or girl in their own estimation, and who that is a man does not feel himself degraded under the consciousness that he is wearing a dirty shirt? The wife who is worthy of the name will never allow these things if she is provided with means for their prevention, and it is in the noble endeavor to maintain for herself and family a respectability of appearance which their station demands, with means and help far too limited, which so irritates, and chafes, and annoys her proper pride, that many a time the wife's heart, and constitution, and health are all broken together. This is the history of multitudes of farmers' wives, and the niggardly natures which allow it, after taking an intelligent view of the subject, are simply beneath contempt. What adds to the better appearance of the person, elevates; what adds to the better appearance of a farm, increases its value and the respectability of the cccupant; so that it is always a good investment, morally and pecuniarily, for a farmer to supply his wife generously and cheerfully, according to his ability, with the means of making her family and home neat, tasteful, and tidy. A dollar's worth of lime, a shilling ribbon, or a few pennies' worth of paint may be so used as to give an impression of life, of cheerfulness, and of thrift about a home altogether beyond the value of the means employed for the purpose.

Finally, let the farmer always remember that his wife's cheerful and hearty coöperation is essential to his success, and is really of as much value in attaining it, all things considered, as any thing that he can do; and as she is very certainly his superior in her moral nature, it legitimately follows that he should not only regard her as his equal in material matters, but should habitually accord to her that deference, that consideration, and that high respect which is of right her due, and which can never fail to impress on the children and servants, who daily witness it, a dignity and an elevation of manner, and thought, and feeling, and deportment which will prove to all who see them that the wife is a lady and the husband a man, a gentleman; and a large pecuniary success, with a high moral position and wide social influence, will be the almost certain results.

